# Imaging of Pulmonary Post-Tuberculosis Sequelae

**DOI:** 10.12669/pjms.36.ICON-Suppl.1722

**Published:** 2020-01

**Authors:** Rafeah Khan, Nuzhat Irfan Malik, Abdul Razaque

**Affiliations:** 1 Dr. Rafeah Khan, Consultant Radiologist, The Indus Hospital, Karachi, Pakistan; 2 Dr. Nuzhat Irfan Malik, Specialist Radiologist, The Indus Hospital, Karachi, Pakistan; 3 Dr. Abdul Razaque, Specialist Radiologist, The Indus Hospital, Karachi, Pakistan

**Keywords:** Tuberculosis, Post TB Sequelae

## Abstract

Worldwide, tuberculosis (TB) is one of the top 10 causes of death, and the leading cause from a single infectious agent. Pakistan has an overwhelming burden of TB and it is a major health hazard for the majority of the rural population. The lung continues to be the most common site of involvement and even after completion of treatment residual changes remain which may affect quality of life. Complications of TB after treatment completion can often be misinterpreted for other active diseases so it is important to recognize and understand the radiologic manifestations of the thoracic sequelae. Post TB sequelae can be categorized into parenchymal, airway disease, pleural/chest wall, vascular and mediastinal. These residual changes can be minor however, some can be debilitating and even fatal.The purpose of this pictorial review is to show the spectrum of residual changes seen on chest radiography and/or computed tomography that persist after treatment completion and bacteriological cure.

## INTRODUCTION

The learning objectives of this study include the following:

- To understand the meaning and clinical importance of pulmonary post tuberculosis (TB) sequelae
- To understand the crucial role of imaging in detecting pulmonary post TB sequelae whether primary or secondary after treatment completion and bacteriological cure.

## BACKGROUND

Worldwide, tuberculosis (TB) is one of the top 10 causes of death, and the leading cause from a single infectious agent.^1^ Pakistan has an overwhelming burden of this disease and despite continuous efforts for early detection and treatment, TB still remains one of the major health hazards for majority of the rural population.^1^,^2^ The lung is the most common site of TB involvement and even after completion of treatment, radiological changes remain which can affect the quality of life.^3^

### Post TB Sequelae

These are the anatomical and pathophysiological changes in the chest which are secondary to complications of pulmonary TB, whether primary or secondary, even after completion of treatment and complete bacteriological cure. These changes may result in pulmonary dysfunction which can vary from minor abnormalities to severe breathlessness, increasing the risk of death from respiratory causes.

More fulminant course is expected in an immunocompromised host.^4^ Therefore, various forms of sequelae and complications may result from both primary and post primary pulmonary TB. It is important to recognize these complications so as not to misinterpret them as ongoing active diseases.

### How common is Post TB Sequelae?

An observational study by Gohar Ali et al in Pakistan for post TB sequelae in patients treated successfully for TB showed that out of 155 patients only 11 (9%) patients had complete resolution of the disease whereas 91% patients developed post treatment parenchymal and pleural sequelae.^5^ These results show that post TB sequelae is very common and should be diagnosed post treatment to enable early intervention for proper management. Pulmonary TB with parenchymal involvement is the most common form of TB.^6^ Therefore, it is not surprising to observe that the maximum number of residual lesions affect the parenchyma. All images included in this manuscript are from patients registered at The Indus Hospital, Karachi, Pakistan.

### Findings and Procedure:

***Role of Imaging:***Laboratory testing for detection of active primary or secondary TB remains the gold standard with Gene Xpert assay now taking precedence over Acid Fast Bacilli smear and culture due to its increased rapidity and sensitivity.^7^,^8^ Radiology plays an assisting role for detection of active tuberculous infection in correlation with laboratory results and strong clinical contact. On the other hand role of imaging for post TB sequelae is pivotal for diagnosis of what could easily be misinterpreted as another pathology. Radiologically, post TB sequelae can be categorized into parenchymal, airway disease, pleural/chest wall, vascular and mediastinal in both adults and children. Table-I gives a detailed categorization of these.

**Table I:** Categorization of complications of pulmonary tuberculosis.

Parenchymal	Airway Disease	Pleural/Chest Wall	Vascular	Mediastinal
Tuberculoma	Bronchiectasis	Empyema	Rassmusen aneurysm	Calcified lymph nodes
Thin walled cavity	Tracheobronchial stenosis	Fibrothorax	Arteritis and thrombosis	Fibrosing mediastinitis
Cicatrization collapse	Broncholithiasis	Bronchopleural fistula	Dilated bronchial arteries	Pericardial TB
Aspergilloma		Pneumothorax		
Bronchogenic CA				

### Parenchymal

**Fig.1:**
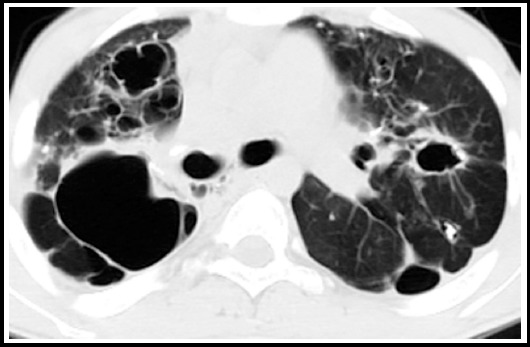
CT chest with IV contrast. Lung and mediastinal windows show tuberculoma in left upper lobe.

***Tuberculoma:*** These may be a manifestation of either primary or postprimary TB. These present radiologically as rounded soft tissue densities which are rarely seen on chest X-ray but are easily visualised on CT. They may be solitary or multiple with wide range of size from a few millimeters to more than 4 cm. They usually have smooth margins with calcification. Fig.-1

**Fig.2:**
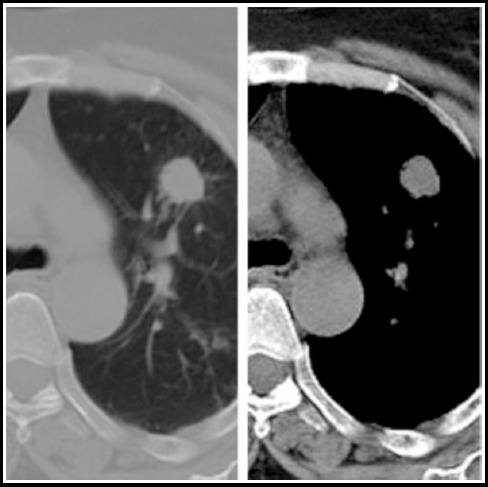
HRCT sections with lung window settings show bilateral cysts with largest thin walled cyst in the right upper lobe.

***Thin walled cavity:*** These may be seen in both active and latent TB. The wall of a chronic cavity varies from 1 mm to 1 cm thickness and may be smooth. It can be difficult to distinguish true cavities from bullae, cysts, or pneumatoceles. Fig-2.

**Fig.3:**
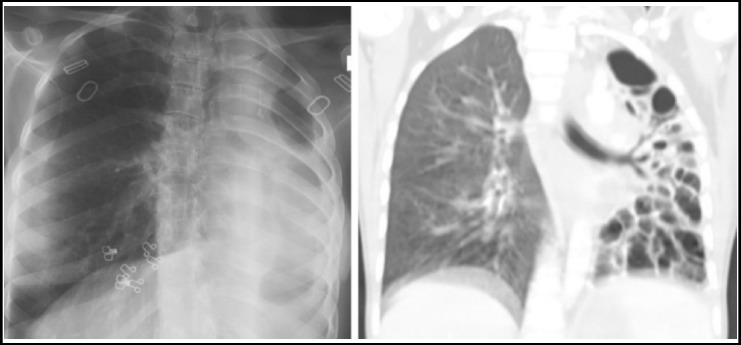
Chest X ray shows loss of lung volume with multicystic area on the left along with ipsilateral mediastinal shift. Corresponding CT chest with lung window settings of the same patient shows cicatrization collapse of the left lung.

***Cicatrization collapse:*** This results from a combination of parenchymal and airway involvement. Part or whole of the lung completely destructs with resulting cavitation which can end in debilitating fibrosis seen as end result in primary or secondary TB. Radiologically there is an area of alveolar destruction with loss of lung volume and ipsilateral mediastinal shift. It is more often unilateral and once the lung is destroyed, it is hard to rule out active disease on imaging alone.Fig-3.

**Fig. 4:**
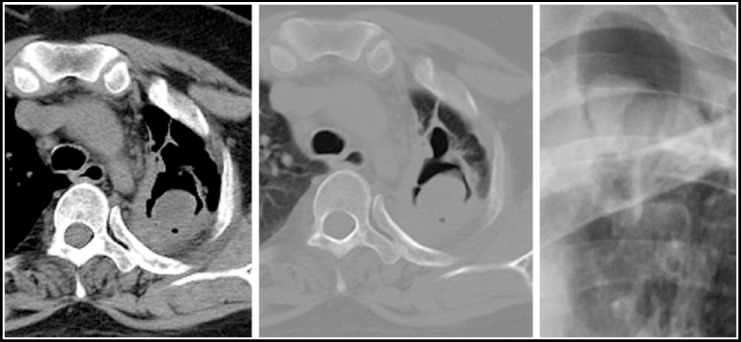
Chest X ray shows a cystic lesion with internal soft tissue opacity and air crescent sign. Corresponding CT chest sections in mediastinal and lung window settings shows a thin walled cavity with a fungal ball.

***Aspergilloma:*** The residual tuberculous cavities post treatment may be a source of colonization of superadded organisms such as fungal ball. This presents radiologically as the ‘air crescent sign’ with a soft tissue density in the cavity which moves with change of body position. It may be asymptomatic or present with hemoptysis. Fig-4.

**Fig.5:**
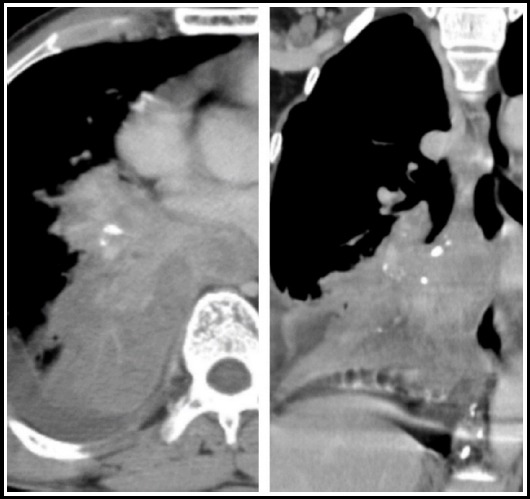
CT scan chest in mediastinal window settings shows an abnormally enhancing, spiculated infraright hilar mass lesion with specks of calcification with adjacent collapse. This patient received ATT 5 years back and now has biopsy proven bronchogenic carcinoma.

***Bronchogenic Carcinoma:*** Although rare, it may develop in tuberculous scar tissue. It may also be a parallel finding along with pulmonary TB or it may be an underlying cause for reactivation of latent TB due to the immunocompromised status of the patient. Radiolographically it manifests as an abnormally enhancing soft tissue mass lesion which may present on the background of old TB changes. Fig-5.

### Airway Disease:

**Fig.6:**
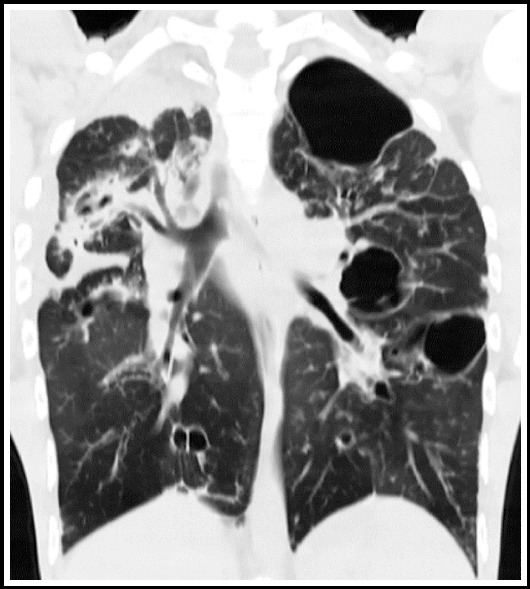
CT scan chest in lung window settings, shows traction bronchiectasis on the right along with patchy fibrosis. At least 3 lung cysts are seen on the left.

***Bronchiectasis:*** This is a common finding in post TB sequelae and it occurs due to destruction and fibrosis of the lung parenchyma with irreversible secondary bronchial dilatation.^9^ It can occur with both primary or secondary exposure. Bronchiectasis located in upper lobe is highly suggestive of a tuberculous origin.^10^ Fig-6.

***Tracheobronchial stenosis:*** Granulomatous changes in the tracheobronchial wall or extrinsic pressure from enlarged peribronchial lymph nodes may start as simple edema and subsequently progress to fibrosis with narrowing of tracheobronchial diameter. The left main bronchus is more frequently affected. Tracheao-bronchial wall thickening, enhancement, and enlarged adjacent mediastinal nodes are common findings on CT.

**Fig.7:**
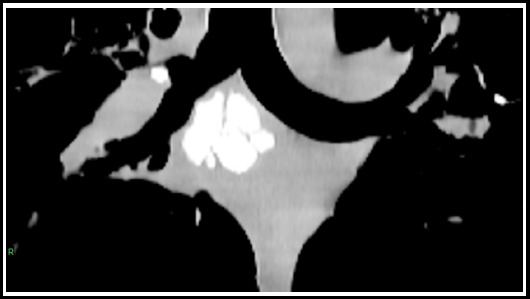
CT scan chest in mediastinal window setting shows multiple calcified subcarinal nodes. A small calcified node is seen adjacent to the main right bronchus which can erode the bronchus and result in a broncholith.

***Broncholithiasis:*** Although uncommon this results from erosion of calcified focus of an adjacent lymph node into the tracheobronchial walls with resulting obstruction. Radiographic manifestations include a change in the position or disappearance of a calcific focus on serial radiographs or development of airway obstruction. CT shows a calcified endo or perinronchial lymph node associated with findings of bronchial obstruction, such as atelectasis, obstructive pneumonitis, or bronchiectasis.Fig-7.

### Pleural/Chest Wall:

**Fig.8:**
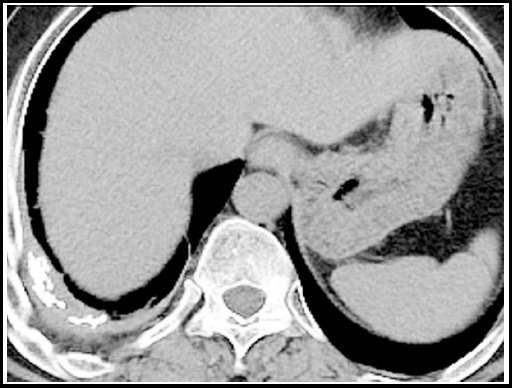
CT scan chest in mediastinal window settings shows right pleural thickenng with calcification.

***Pleural thickening with calcification:*** Tuberculous pleuritis usually resolves completely even in the absence of treatment, however, in some patients, chronic complications occur during the healing of the TB lesions, or appear as late sequelae such as pleural thickening and pleural calcification. It is usually asymptomatic but may also present with discomfort while breathing. CT shows pleural thickening and specks of calcification which are usually focal and unilateral but may be bilateral. Fig-8.

**Fig.9:**
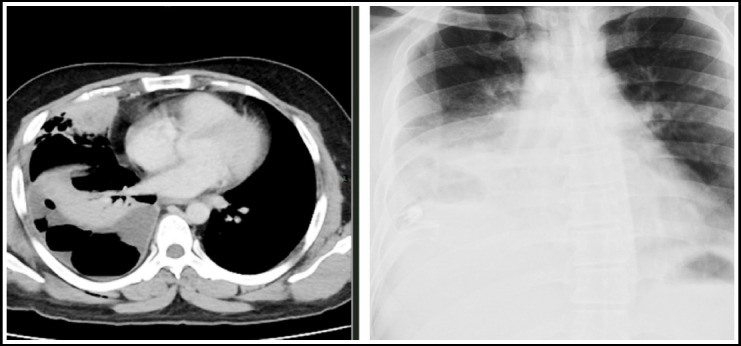
Chest X ray shows right pleural thickening with haze in the right lower lobe. Corresponding CT scan chest with IV contrast shows high density pleural effusion containing air. Diagnostic pleural tap showed frank pus.

***Empyema:*** Persistent purulent fluid, caused by rupture of a subpleural caseous focus, may be seen in the pleural space even after bacteriological cure and this fluid is difficult to culture. CT findings would include nodular, thickened and enhancing pleura with high density pleural fluid. Chyliform effusion is also a known post TB pleural complication. CT findings would include fat-fluid or fat-calcium level in the pleural cavity. Fig-9.

**Fig.10:**
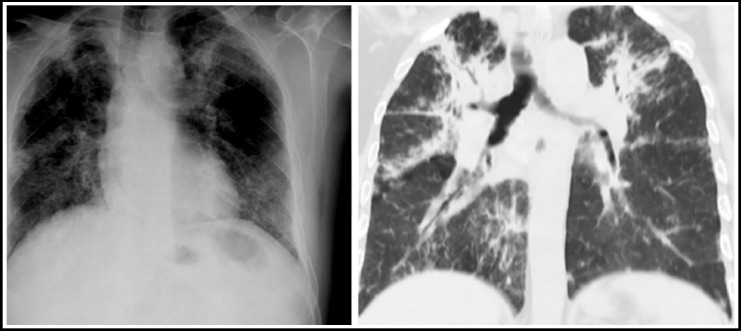
Chest X ray shows increased interstitial markings bilaterally. Corresponding HRCT shows fibrous changes in both lung fields consistent with fibrothorax.

***Fibrothorax:*** This is a medical condition characterised by scarring (fibrosis) of the pleural space surrounding the lungs that is severe enough to cause reduced movement of the lung and ribcage. On CT it manifests as diffuse pleural thickening but without effusion suggesting inactivity. Patients with diffuse pleural thickening should be closely monitored for the development of restrictive lung disease with serial pulmonary function tests. Fig-10.

**Fig.11:**
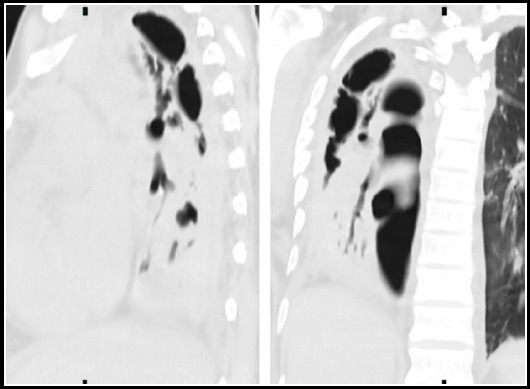
CT chest in lung window settings shows right sided pneumothorax which appears to be communicating with right sided bronchi consistent with bronchopleural fistula.

***Bronchopleural fistula:*** When associated with TB it may be either spontaneous, secondary to trauma or a surgical procedure and is associated with a high mortality. This can be seen after successful treatment and the diagnosis is based on an increasing amount of sputum production, air in the pleural space and a changing air-fluid level seen on chest X ray. CT scan is useful to visualize the communication between the pleural space and the airway. Fig-11.

**Fig.12:**
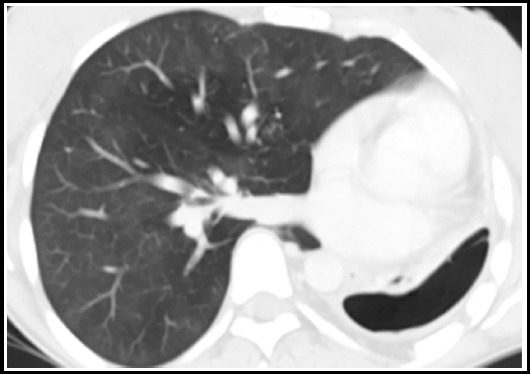
CT scan section in lung window settings, shows loculated left pneumothorax with loss of lung volume on the left. There is compensatory hypertrophy of the right lung field.

**Fig.13:**
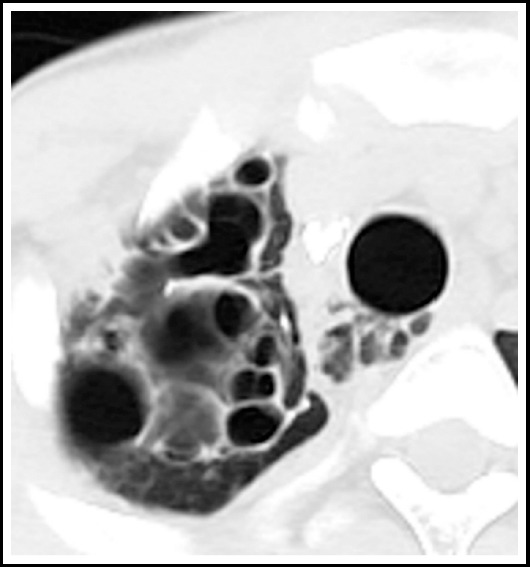
CT scan chest section in lung window setting shows loculated pneumomediastinum secondary to chronic cough. Saccular bronchiectatic changes are seen in the right upper lobe.

***Pneumothorax:*** This usually results from rupture due to liquefactive necrosis of the pleura secondary to the caseous infiltrate. Secondary pneumothorax is a recognized complication of postprimary TB.^11^ Tube drainage is the treatment of choice. Radiographic findings could include air in pleural cavity with severe cavitatory changes in the underlying lung. Fig-12 and 13.

**Fig.14:**
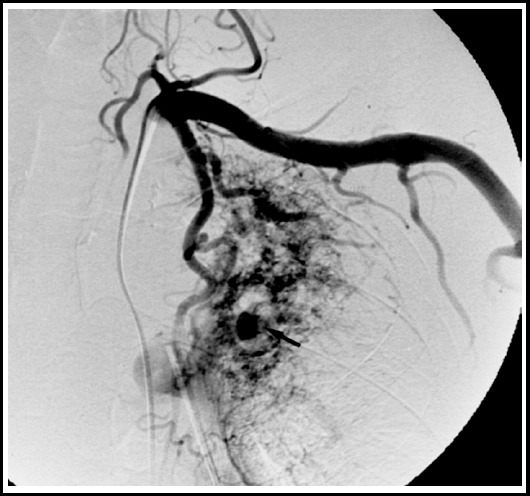
Angiogrpahy film shows a rasmeussen aneurysm in the right upper lobe in a post TB patient who presented with hemoptysis.

***Vascular Complications:*** Bronchial artery enlargement accompanying bronchiectasis is difficult to assess on a plain unenhanced study, however, tubular structures in the perihilar region not taking the shape of normal vessels should be further investigated for vascular complications. Vasculitis or thrombosis are features more suggestive of active disease. Rasmussen aneurysm, although rare, is caused by weakening of the pulmonary artery wall from granulation tissue which gradually gets replaced by fibrin. Resulting pseudoaneurysm formation may cause vessel wall rupture. Hemoptysis is the usual presenting symptom and may be life-threatening. Fig-14.

### MEDIASTINAL:

**Fig.15:**
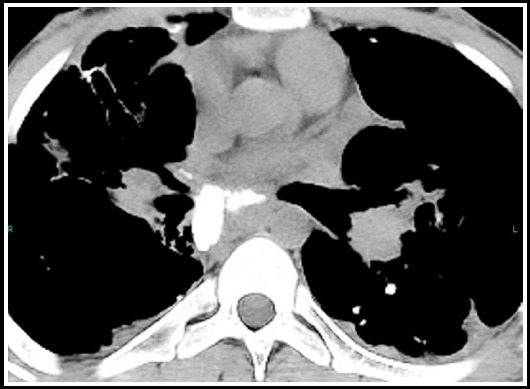
CT scan section in mediastinal window setting, shows calcified mediastinal lymph nodes.

***Calcified lymph nodes:*** Mediastinal lymph node involvement is common in primary TB with incidence being the highest during childhood which decreases with increasing age.^12^ After successful treatment completion the previously centrally necrotic nodes may either disappear or form a residual mass composed of fibrotic tissue and calcifications. These are asymptomatic but may indent upon adjacent structures. Fig-15.

### Fibrosing mediastinitis

**Fig.16:**
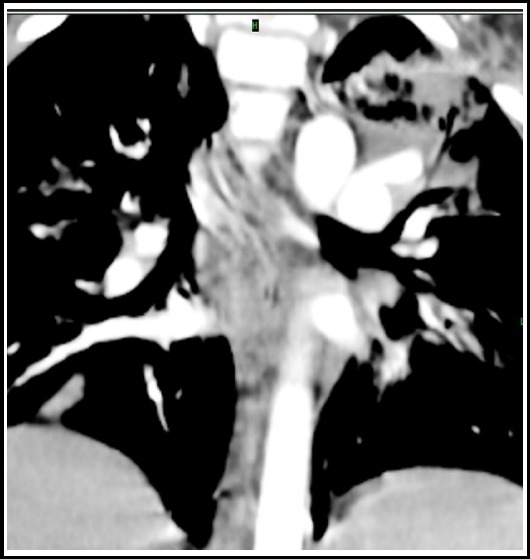
CT scan with IV contrast section in mediastinal window setting shows mediastinal fibrosis secondary to TB.

This is caused by excessive fibrosis in the mediastinum and it progresses insidiously with symptoms ranging from mild to severe from compression over the superior vena cava, esophagus and central airway. The radiographic findings include mediastinal widening or a localized mass. CT shows calcified mediastinal or hilar mass with central airway narrowing, pulmonary vessel encasement and compression of superior vena cava. Bronchial obstruction secondary to fibrosis may result in obstructive pneumonia or atelectasis. Fig-16.

**Fig.17:**
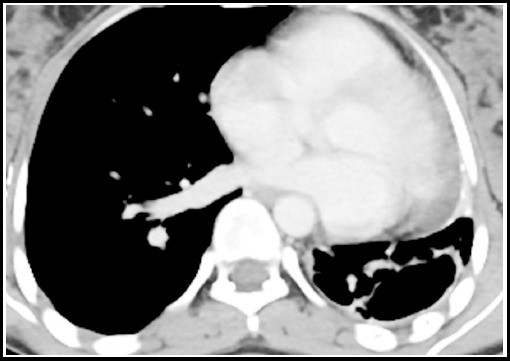
CT scan with IV contrast section shows pericardial thickening with calcification. Streak of pericardial effusion is also seen.

***Pericardial TB*** Pericardial involvement is very rare and usually results from tuberculous lymphadenitis extension because of the close anatomic relationship of the lymph node with the posterior pericardial sac. Fibrous or calcific thickening of the pericardium results in constrictive pericarditis and occurs in about 10% of patients with tuberculous pericarditis.^13^ CT shows pericardial thickening of more than 3 mm with or without pericardial effusion. Fig-17.

## CONCLUSION

Post TB sequelae can either be symptomatic or asymptomatic but it is imperative to be able to detect these residual changes so as to rule out other active diseases.

### Authors’ Contribution

**RK:** Conceived, designed and wrote the manuscript.

**NIM and AR:** Did data collection, review and final approval of manuscript.
